# Lamina Propria Phagocyte Profiling Reveals Targetable Signaling Pathways in Refractory Inflammatory Bowel Disease

**DOI:** 10.1016/j.gastha.2022.01.005

**Published:** 2022-03-30

**Authors:** Gillian E. Jacobsen, Irina Fernández, Maria A. Quintero, Ana M. Santander, Judith Pignac-Kobinger, Oriana M. Damas, Amar R. Deshpande, David H. Kerman, Yuguang Ban, Zhen Gao, Tiago C. Silva, Lily Wang, Ashley H. Beecham, Jacob L. McCauley, Juan F. Burgueño, Maria T. Abreu

**Affiliations:** 1 Department of Microbiology and Immunology, Miller School of Medicine, University of Miami, Miami, Florida; 2 Division of Gastroenterology, Department of Medicine, Miller School of Medicine, University of Miami, Miami, Florida; 3 Medical Scientist Training Program, Miller School of Medicine, University of Miami, Miami, Florida; 4 Biostatistics and Bioinformatics Shared Resource, Sylvester Comprehensive Cancer Center, University of Miami, Miami, Florida; 5 Division of Biostatistics, Department of Public Health Sciences, Miller School of Medicine, University of Miami, Miami, Florida; 6 John P. Hussman Institute for Human Genomics, Miller School of Medicine, University of Miami, Miami, Florida

**Keywords:** IBD, RNA Sequencing, Phagocytes, Anti-TNF, JAK-STAT

## Abstract

**BACKGROUND AND AIMS:**

Lamina propria phagocytes are key mediators of inflammatory bowel disease (IBD). We aimed to understand the transcriptomic and functional differences in these cells based on location, disease type, inflammation state, and medication use in patients with IBD.

**METHODS:**

Phagocytic immune cells in the lamina propria, as defined by the marker CD11b, were isolated from 54 unique patients (n = 111 gut mucosal biopsies). We performed flow cytometry for cell phenotyping (n = 30) and RNA sequencing with differential gene expression analysis (n = 58). We further cultured these cells in vitro and exposed them to janus kinase inhibitors to measure cytokine output (n = 27). Finally, we matched patient genomic data to our RNA sequencing data to perform candidate gene expression quantitative trait locus analysis (n = 34).

**RESULTS:**

We found distinct differences in gene expression between CD11b^+^ cells from the colon vs ileum, as well as in different inflammatory states and, to a lesser degree, IBD types (Crohn’s disease or ulcerative colitis). These genes mapped to targetable immune pathways and metabolic and cancer pathways. We further explored the janus kinase-signal transducer and activator of transcription pathway, which was upregulated across many comparisons including in biopsies from anti–tumor necrosis factor refractory patients. We found that isolated CD11b^+^ cells treated with janus kinase inhibitors had decreased secretion of cytokines tumor necrosis factora and interleukin-8 (*P* ≤ .05). We also found 3 genetic variants acting as expression quantitative trait loci (*P* ≤ .1) within our CD11b^+^ data set.

**CONCLUSIONS:**

Lamina propria phagocytes from IBD mucosa provide pathogenetic clues on the nature of treatment refractoriness and inform new targets for therapy.

## Introduction

Inflammatory bowel diseases (IBD), including Crohn’s disease (CD) and ulcerative colitis (UC), have increasing incidence and prevalence in the developing world and immigrant populations such as Hispanics within the United States.^1^ IBD is characterized by chronic inflammation of the gut in response to commensal microbiota.^2,3^ Understanding of IBD pathogenesis has been enhanced by genome-wide association studies, which have identified greater than 240 loci significantly associated with IBD.^4^ These loci have unveiled relevant pathways in IBD pathophysiology that reflect interactions between the host and the microbiome, including genes involved in microbial recognition and clearance, shaping of the adaptive immune response, and epithelial integrity. In the lamina propria, microbial sensing, autophagy, and priming of the adaptive immune response converge in phagocytes.^2,5,6^ Phagocytes act as sentinels, sampling the luminal environment, killing invading bacteria, processing them, and presenting antigens to T cells.^5,6^ Thus, lamina propria phagocytes along with epithelial cells represent a first line of defense and play a balancing act between tolerance toward commensal microbes and generation of immune responses toward pathogenic microorganisms.

Inappropriate responses by lamina propria phagocytes have been linked to IBD.^2^ Both dendritic cells and macrophages from patients with IBD display altered cell markers and cytokine production when compared with non-IBD controls.^7,8^ Furthermore, bacterial clearing defects have been demonstrated in phagocytes of patients with IBD.^3^ However, most reports addressing the function of phagocytes have focused on circulating dendritic cells, monocytes, or monocyte-derived macrophages,^9–11^ rather than on resident phagocyte populations located in the lamina propria. We have shown that 16S rDNA sequencing within lamina propria–isolated CD11b^+^ phagocytic cells reveals a deeper level of dysbiosis than mucosa-associated microbiota in IBD biopsies, with a greater representation of Proteo-bacteria.^6^ We also used a targeted Nanostring approach and found that gene expression in CD11b^+^ cells from inflamed areas expressed higher levels of inflammatory genes than that from uninflamed areas, including *S100A8/9*, *CXCR1*, *CXCR2*, *OSM*, *LILRB3*, *IL1B*, *CXCL1*, *CXCL8*, and others.^6^ These data suggest that studying the lamina propria CD11b^+^ innate immune cell population may provide insight into inflammatory pathways not targeted previously.

In the present study, we used RNA sequencing to examine gene expression in macrophages, neutrophils, and dendritic cells. We isolated these phagocytes from biopsy specimens of patients with CD and UC using a common marker among these cells, CD11b. We tested the hypothesis that lamina propria CD11b^+^ cells from inflamed and uninflamed areas of patients with IBD express distinct gene signatures and altered cytokine production. We further hypothesized that medication exposure and medication refractoriness would be reflected in gene expression and could be used to identify new targets for therapy. Finally, we aimed to incorporate whole genomic sequencing data from our patients to determine if any differentially expressed (DE) genes were associated with genetic variants in IBD risk loci. Our data highlight basic pathogenetic mechanisms of refractory IBD and potential avenues for combination therapy.

## Results

### Granulocytes Are the Largest Subpopulation of Lamina Propria CD11b^+^ Phagocytes in Active IBD

**Figure 1.**
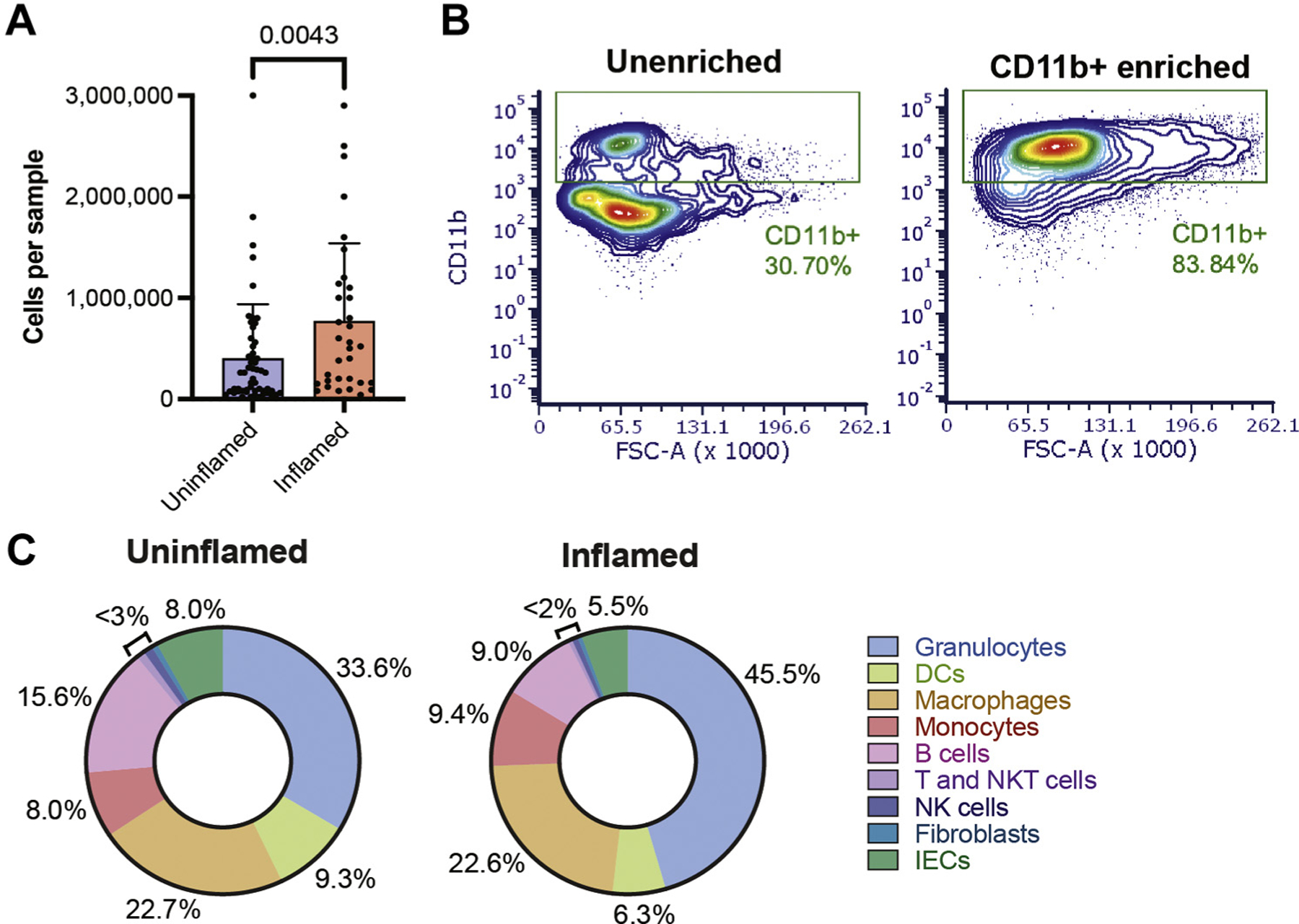
Characterization of CD11b^+^-enriched cells shows an increased overall cell count and granulocyte percentage in inflamed biopsies. (A) After magnetic column sorting, lamina propria CD11b^+^-enriched cells from histologically uninflamed (n = 54) and inflamed biopsies (n = 33) were counted via a hemocytometer. Uninflamed biopsies yielded an average of approximately 400,000 cells and inflamed biopsies, approximately 750,000 cells (Mann-Whitney U-test *P =* .0043). (B) Flow cytometry gating of CD11b^+^ cells in a representative unenriched sample (all lamina propria cells) and a CD11b^+^-enriched sample, after gating on live, single, CD45^+^ cells. (C) CD11b^+^-enriched cells from histologically uninflamed (n = 20) and inflamed biopsies (n = 10) were phenotyped via flow cytometry. Mean percentages of each cell type are shown.

Biopsies (ileum and colon) were collected prospectively in 54 patients (Table 1). A large proportion of these patients were refractory to IBD medications; of the 59% of patients currently on biologics, 72% still exhibited inflammation in one or more biopsied areas (Table 2). In addition, 41% of patients reported using at least one other biologic in the past. A total of 111 biopsy samples were collected and categorized as inflamed or uninflamed based on endoscopy and histology (Table 2). After enriching for CD11b^+^ cells, we found that inflamed tissue had an increased number of cells compared with uninflamed tissue (average approximately 750,000 vs 400,000 cells per sample; Mann-Whitney U-test *P =*.0043) (Figure 1A). We used flow cytometry to confirm CD11b^+^ phagocyte enrichment (Figure 1B). The CD11b^+^ phagocytic cell population included mostly granulocytes (CD66^+^), macrophages (CD14^+^, CD31^−^), monocytes (CD14^+^, CD31^+^), and dendritic cells (CD1c^+^) (Figure 1C). In addition, a small proportion of B cells (CD11b^+^, CD19^+^), intestinal epithelial cells (CD45^−^, Epcam/CD326^+^), and fibroblasts (CD45^−^, S100A4^+^) were also present. Inflamed biopsies had an increased percentage (inflamed 45 ± 9%, uninflamed 34 ± 10%; unpaired t-test *P =* .0665) and absolute number of granulocytes, which include neutrophils. Neutrophilic infiltration is a major indicator of IBD activity and may be critically linked to ongoing inflammation.^12^ These data demonstrate that CD11b^+^ enrichment reflects the inflammatory state of the biopsies.

### Lamina Propria CD11b^+^ Cell Transcriptional Profiles Differ Greatly Between the Ileum and the Colon and Relate to Innate Immunity and Metabolic Pathways

**Figure 2.**
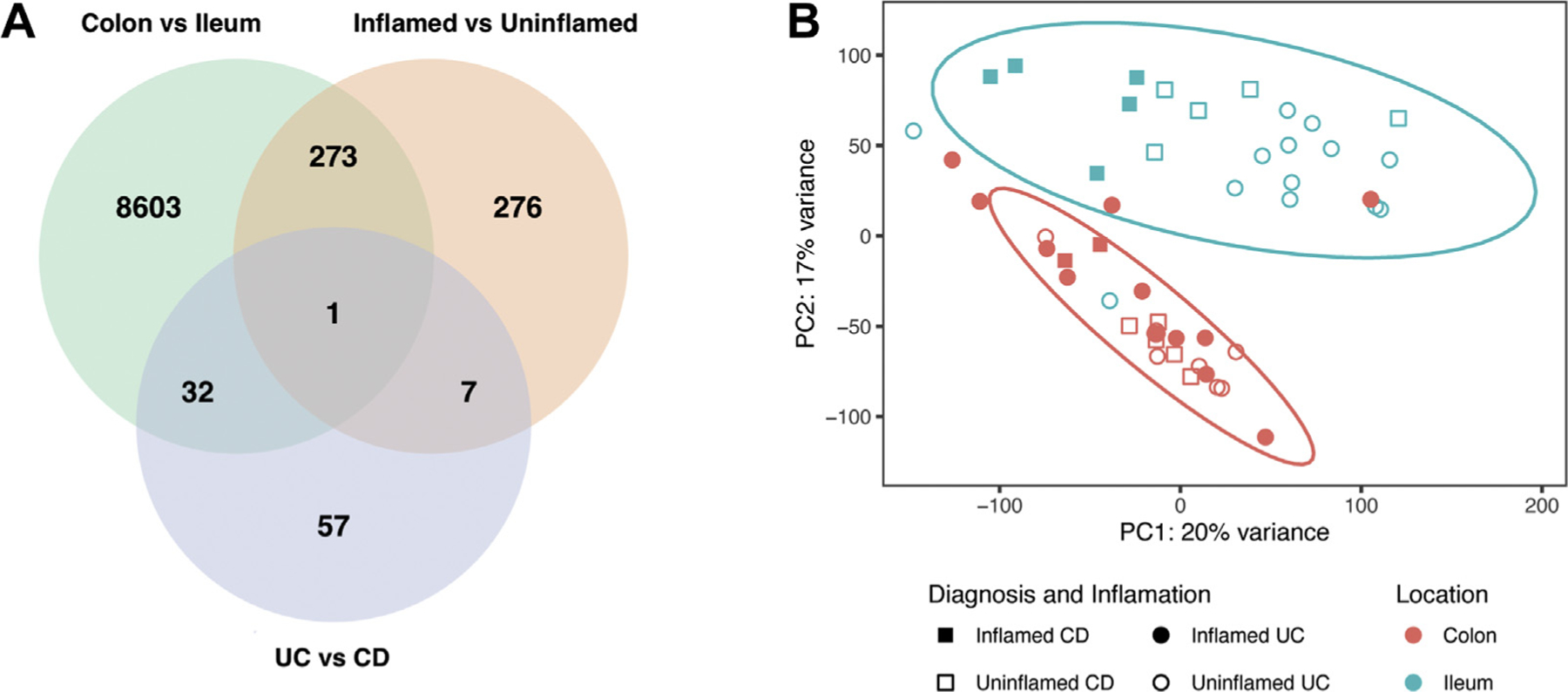
CD11b^+^ cell transcriptional profiles show largest differences based on location. (A) Venn diagram of the number of significant (*P ≤* .05) DE genes in CD11b^+^ cells across 3 global comparisons: colon vs ileum, UC vs CD, and inflamed vs uninflamed. The one differentially expressed gene common among all comparisons (center) was *NTS*. (B) Principal component analysis (PCA) of all samples (n = 58) based on the transcriptional profiles showed separation primarily by location. Ileum samples showed additional grouping by inflammation and diagnosis.

To define the gene signatures that characterize inflammation in patients with IBD, we performed RNA sequencing on isolated lamina propria CD11b^+^ cells. To preserve neutrophilic signatures, we performed bulk RNA sequencing.^13^ We first looked broadly at differential gene expression by comparing sample location (ileum vs colon), type of disease (UC vs CD), and inflammatory state (inflamed vs uninflamed) (Figure 2A). The highest number of DE genes occurred between the colon vs ileum: a total of 8909 significant DE genes, with 4112 genes upregulated in the colon and 4797 genes upregulated in the ileum (false discovery rate ≤0.05). By comparison, fewer yet still relevant differences were seen between UC vs CD gene expression (98 DE genes) or between inflamed vs uninflamed gene expression (558 DE genes). We assessed the impact of potential confounders of gene expression including age, gender, race, and years of disease but did not find a significant contribution of these factors (Table A3). Overall principal component analysis of our data set showed distinct grouping of samples by location, as well as less-distinct grouping by diagnosis and inflammation within ileum samples (Figure 2B). Owing to the substantial differences based on location, we hereafter stratified all other comparisons by location first.

**Figure 3.**
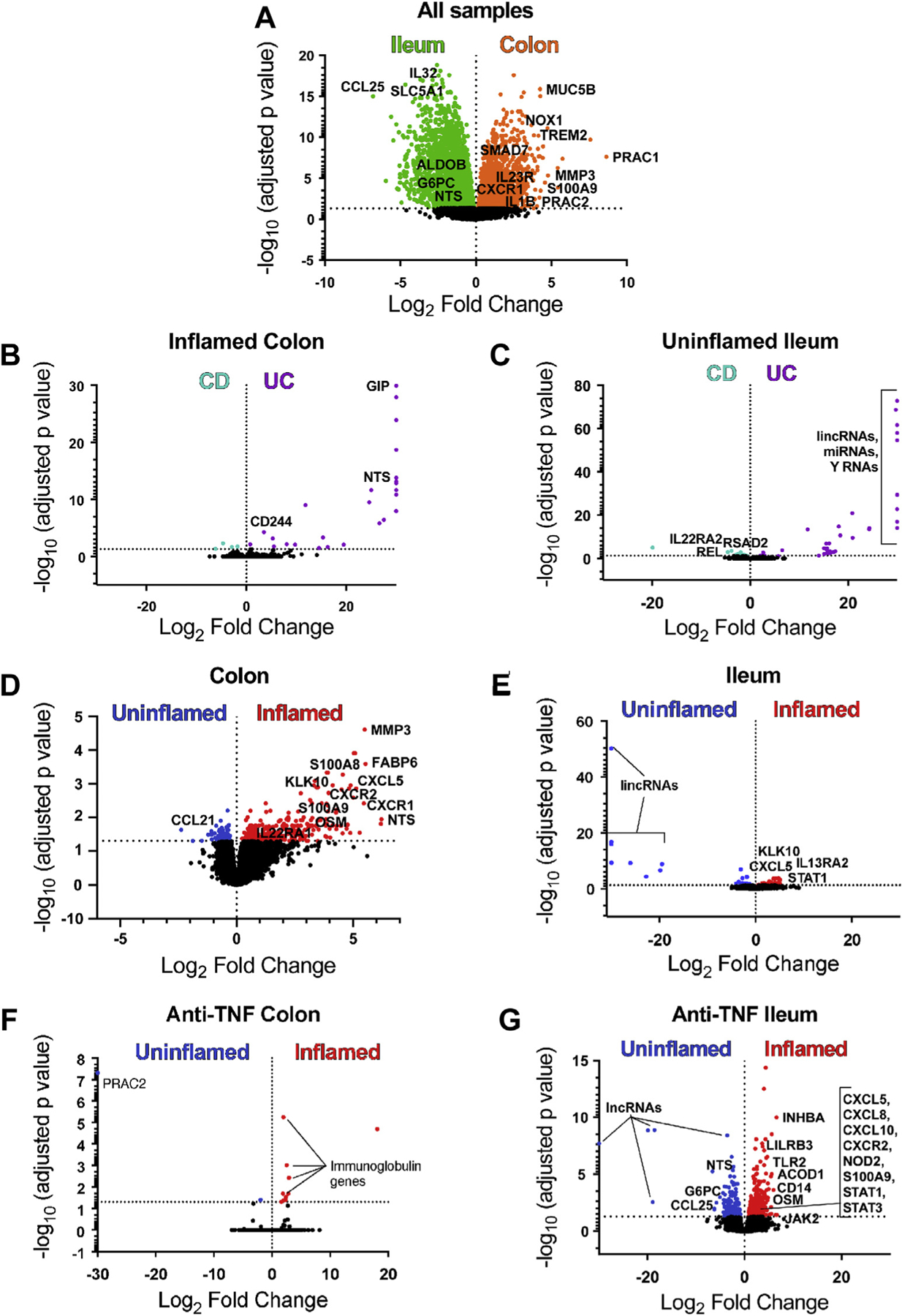
CD11b^+^ cell differential gene expression analysis across multiple sample comparisons. CD11b^+^ cells showed differential gene expression between (A) all samples, ileum vs colon (n = 58), (B) inflamed colon, CD vs UC (n = 15), (C) uninflamed ileum, CD vs UC (n = 21), (D) colon, uninflamed vs inflamed (n = 32), (E) ileum, uninflamed vs inflamed (n = 26), (F) anti-TNF colon, uninflamed vs inflamed (n = 8), and (G) anti-TNF ileum, uninflamed vs inflamed (n = 8). Colored genes were significantly differentially expressed (*P* ≤ .05).

Looking further into DE genes in the colon vs ileum, we found that most IBD inflammation-associated^14,15^ genes were higher in the colon, such as *IL1B*, *IL23R*, *CXCR1*, and *S100A8/S100A9* (calprotectin) (Figure 3A). In contrast, ileum samples were higher in metabolic genes such as *ALDOB* (aldolase, fructose-bisphosphate B), *G6PC1* (glucose-6-phosphatase catalytic subunit 1), and *SLC5A1* (solute carrier family 5 member 1). *NTS* (neurotensin) was also higher in the ileum; in fact, *NTS* was the only DE gene across all 3 major comparisons (Figure 2A). Although neurotensin was originally discovered in the central nervous system, it has since been found to have proinflammatory,^16^ enteroendocrine,^17^ and metabolic properties^18^ in the gastrointestinal tract. Its upregulation has also been associated with colorectal cancer.^17^

**Figure 4.**
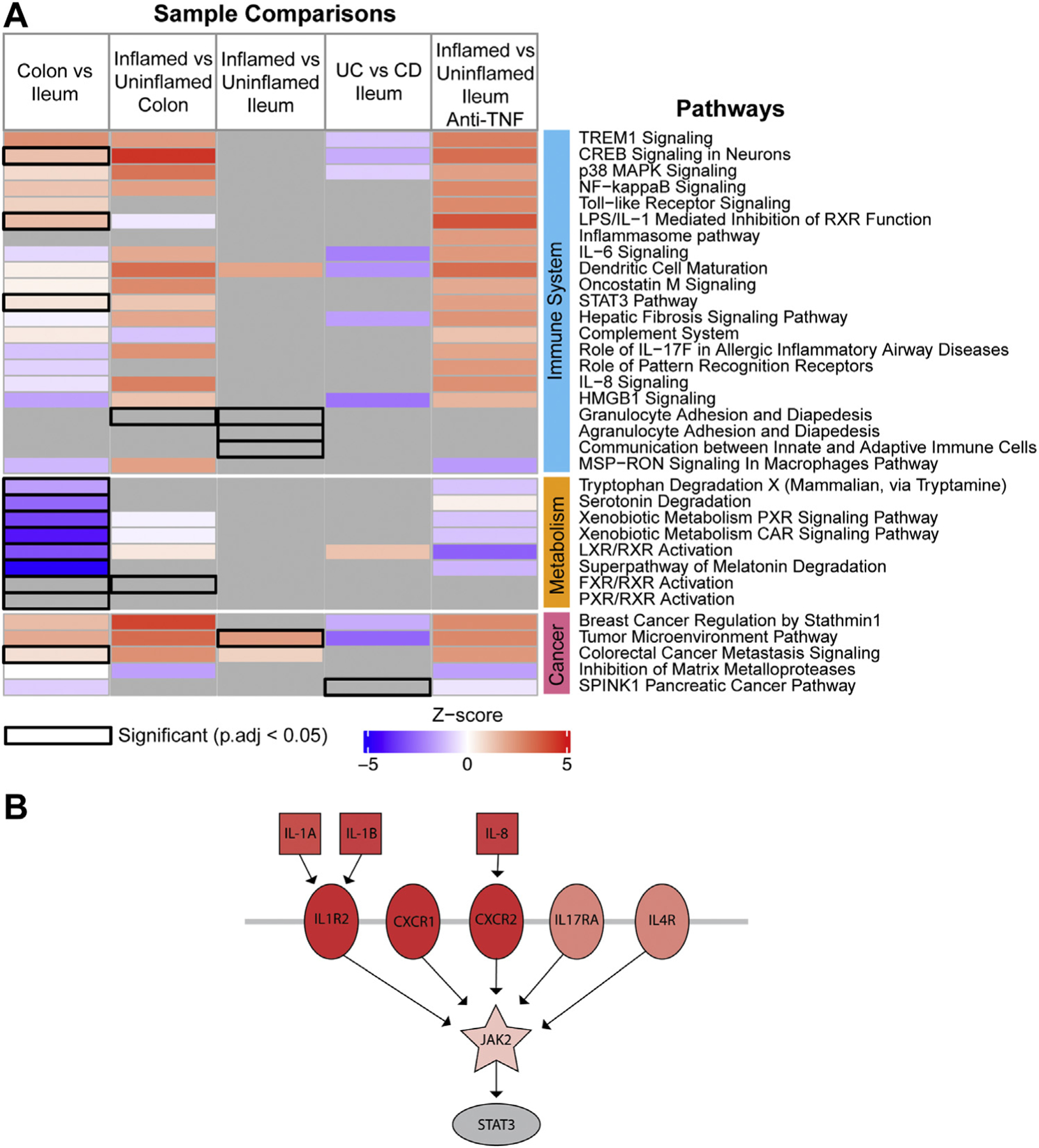
Targetable signaling pathways are upregulated in inflamed and anti-TNF refractory samples. (A) Heatmap of enriched pathways from IPA sorted by the pathway activation z-score for the colon vs the ileum with the border bolded for significant (adjusted *P ≤* .05) enrichment. The title of each column lists the 2 compared groups. A positive (red) z-score indicates pathway upregulation in the first group, whereas a negative (blue) z-score indicates pathway upregulation in the second group. Gray indicates undirected pathway enrichment (the z-score could not be calculated). (B) Simplified upstream regulatory network of the STAT3 pathway in anti-TNF–treated ileum samples. The red color intensity of each gene correlates with a positive z-score (upregulation) in inflamed anti-TNF–treated ileum samples.

We next used Ingenuity Pathway Analysis (IPA) to map pathways driving the large differences between ileal and colonic CD11b^+^ cell signatures. Pathways upregulated in the colon included innate immune pathways such as toll-like receptor signaling, interleukin (IL)-6 and IL-8 signaling, inflammasome, and the complement system (Figure 4A). TREM1 (triggering receptor expressed on myeloid cells 1) signaling, which occurs in neutrophils, monocytes, and macrophages and amplifies inflammatory processes in IBD,^19^ was also upregulated in the colon. In general, more metabolic pathways such as lipid metabolism were upregulated in the ileum. Activation of FXR/RXR, LXR/RXR, and PXR/RXR, which are regulators of bile salt and cholesterol homeostasis, was upregulated in the ileum. Conversely, one of the most significantly enriched pathways in the colon was lipopolysaccharide (LPS)/IL-1–mediated inhibition of RXR (Figure 4A). Tryptophan, melatonin, and serotonin degradation was also highly upregulated in the ileum. These molecules have previously been shown to be altered in IBD compared with healthy controls.^20^ Furthermore, xenobiotic metabolism via CAR/PXR signaling pathways was also increased in the ileum. Recent data demonstrate that bile acids absorbed in the ileum upregulate the xenobiotic transporter MDR1 and protect against inflammation.^21^ Overall, these data suggest that the location or cellular environment plays a marked role in determining the gene expression of phagocytes.

### Lamina Propria CD11b^+^ Cell Genetic Signatures Are Distinct in CD and UC

Although the genetic architecture of UC and CD is known to be similar, the disease phenotypes can be quite disparate, suggesting that local gene expression may be responsible. We therefore examined differences between CD11b^+^ cell gene expression in UC vs CD, stratifying by disease location and inflammation. Although relatively fewer genes were DE, these genes exhibited a much greater fold change between UC and CD than other comparisons (Figure 3B and C). In the colon, we compared inflamed CD samples with inflamed UC samples to investigate gene signatures in active disease. The inflamed CD colon showed upregulated *CD244*, a cell surface receptor typically expressed by natural killer cells that is responsible for granzyme B production and subsequent apoptosis.^22^ The most significant and highly upregulated gene in the inflamed UC colon was *GIP* (glucose-dependent insulinotropic polypeptide), which is increased in obese individuals and involved with chronic low-grade inflammation.^23^ In the ileum, we compared only uninflamed UC and CD samples because UC inflammation is confined to the colon. We found that the CD ileum had more expression of genes involved in regulation of cytokine production, such as *IL22RA2* and *REL* (NF-kB subunit) (Figure 3C). The janus kinase-signal transducer and activator of transcription (JAK-STAT) cascade, for which IL22RA2 is an upstream regulator, is the target of emerging therapies in IBD.^24^ NF-kB–producing macrophages are known to be high in anti–tumor necrosis factor (TNF)-refractory patients with CD.^13^ Pathways upregulated in the CD ileum similarly included inflammatory pathways such as IL-6 signaling and HMGB1 (high-mobility group box 1) signaling (Figure 4A). In contrast, the uninflamed UC ileum showed upregulation of various noncoding RNAs (Figure 3C) as well as LXR/RXR activation, akin to general ileal signatures. Therefore, it appears as though the uninflamed CD ileum shows a shift toward inflammatory or colon-like signatures compared with uninflamed UC ileum signatures.

### Lamina Propria CD11b^+^ Gene Expression Reflects Inflammation State and Medication Refractoriness in IBD

To define the transcriptional alterations associated with inflammation in lamina propria CD11b^+^ cells, we segregated all biopsy samples regardless of the IBD type by inflammation status. Of the patients in our cohort with inflammation, most were on biologics (86%) and/or immunomodulators (29%), implying that inflammation was seen in spite of medical therapy. Looking first at the colon, a total of 484 DE genes were upregulated in inflamed samples, including proinflammatory genes such as *CXCR2*, *CXCL5*, S100A8, S100A9, *OSM*, and *IL22RA1* (Figure 3D). Some of these gene products are already used to monitor active inflammation in IBD, such as calprotectin (*S100A8/9*).^14^ Similarly, upregulated pathways included positive regulation of immune response, immune cell adhesion and diapedesis, communication between innate and adaptive immune cells, and response to bacteria (Figure 4A). A number of upregulated genes were also linked to colorectal cancer, such as *KLK10* (kallikrein-10),^25^ *MMP3* (matrix metalloproteinase 3),^26^ *FABP6* (fatty acid binding protein 6),^27^ and *NTS*^17^ (Figure 4A). In the ileum, we found far fewer DE genes based on inflammation generally (Figure 3E). The inflamed ileum highly expressed *STAT1*, which induces expression of interferon-stimulated genes and is indirectly targeted by new JAK-inhibitor therapies for IBD.^24^ Most genes highly upregulated in the uninflamed ileum were long noncoding RNAs (lncRNAs).^28^ Overall, these data suggest that there are pathways common to inflammation regardless of the IBD type that could be used as markers of inflammation or targets for therapy.

To further investigate the transcriptional signatures of medication refractoriness, we next specifically looked at samples from patients with inflammation despite being on anti-TNF therapy. In the colon, 52 DE genes were significant between inflamed and uninflamed samples on anti-TNFs. These genes were mostly immunoglobulin genes upregulated in the anti-TNF–treated inflamed colon, suggesting that CD11b^+^ B cells may play a role in medication refractoriness (Figure 3F). *PRAC2*, which is known to be expressed in healthy colon tissue, but its relevance in IBD is unclear,^29^ was also high in the anti-TNF–treated uninflamed colon. In the ileum, surprisingly, we found over 500 DE genes by inflammation status when focusing specifically on anti-TNF–treated patients (Figure 3G). These included inflammatory genes such as *STAT1*, *STAT3*, *CXCL5*, *CXCL8*, *CXCL10*, *CXCR2*, *IL1B*, S100A9, and *OSM* (Figure 3G). Metabolic genes were also upregulated in the anti-TNF–treated, inflamed ileum, such as *ACOD1* (aconitate decarboxylase 1), a known regulator of immunometabolism during inflammation and infection.^30^ Meanwhile, in uninflamed samples on anti-TNF therapy, *NTS* was highly upregulated as well as *CCL25* and various lncRNAs. A subset of these DE genes was confirmed with quantitative polymerase chain reaction (Figure A1). We then used IPA to predict disease states and biological functions associated with this anti-TNF-refractory gene expression pattern and found that immune cell migration, inflammatory response, and autoimmunity pathways were significantly enriched (Table A4). Top upstream regulators of these pathways included LPS, interferon-gamma, dexamethasone, IL-10, TNF, IL-1*β*, and immunoglobulins (Table A5). *OSM*, which was upregulated in both the inflamed colon and anti-TNF refractory ileum, is a marker of anti-TNF-resistant disease.^31^ This suggests that the increase in *OSM* seen in anti-TNF nonresponse may originate from CD11b^+^ cells.

### Janus Kinase-Signal Transducer and Activator of Transcription and TREM1 Signaling Pathways Are Upregulated in CD11b^+^ Cells From Inflamed and Anti-TNF-Refractory Samples

One of our goals in using RNA sequencing on CD11b^+^ cells was to identify potentially targetable pathways in IBD in an unbiased approach. One common target seen across multiple comparisons was the JAK-STAT pathway. The STAT3 pathway was highly enriched in colon vs ileum, inflamed colon vs uninflamed colon, and anti-TNF inflamed ileum vs anti-TNF uninflamed ileum, as per IPA (Figure 4A). Looking at anti-TNF inflamed vs uninflamed ileum samples specifically, upstream regulators of the STAT3 pathway include upregulated genes such as *IL1A*, *IL1B*, and *JAK2* (Figure 4B). Other top upstream regulators included *NFKB*, *RELA*, *STAT1*, and again *STAT3* (as per TRRUST^32^; Figure A3). In addition, *JAK2* was involved in 15 of the top 50 upregulated IPA pathways in this comparison (Table A7). Both JAK2 and STAT3 are involved in IL-17 signaling and promotion of Th17 cells, which are strongly implicated in the pathogenesis of IBD.^33,34^

Another pathway of particular interest was TREM1 signaling. As mentioned previously, *TREM1* is expressed by myeloid cells and participates in amplifying inflammation.^35^ In addition, *TREM1* expression in whole blood can predict response to anti-TNF medications.^36,37^ Our IPA results showed an upregulation of TREM1 signaling in the colon vs the ileum and the anti-TNF inflamed ileum vs the uninflamed ileum, as well as the CD ileum vs the UC ileum (Figure 4A). Our data showing *TREM1* upregulated in inflamed samples on anti-TNF therapy (refractory samples) not only substantiate prior research on its predictive capabilities, but also suggest TREM1 as a possible therapeutic target.

### JAK-Inhibitors Dampen Cytokine Release in Gut-Derived CD11b^+^ Cells

**Figure 5.**
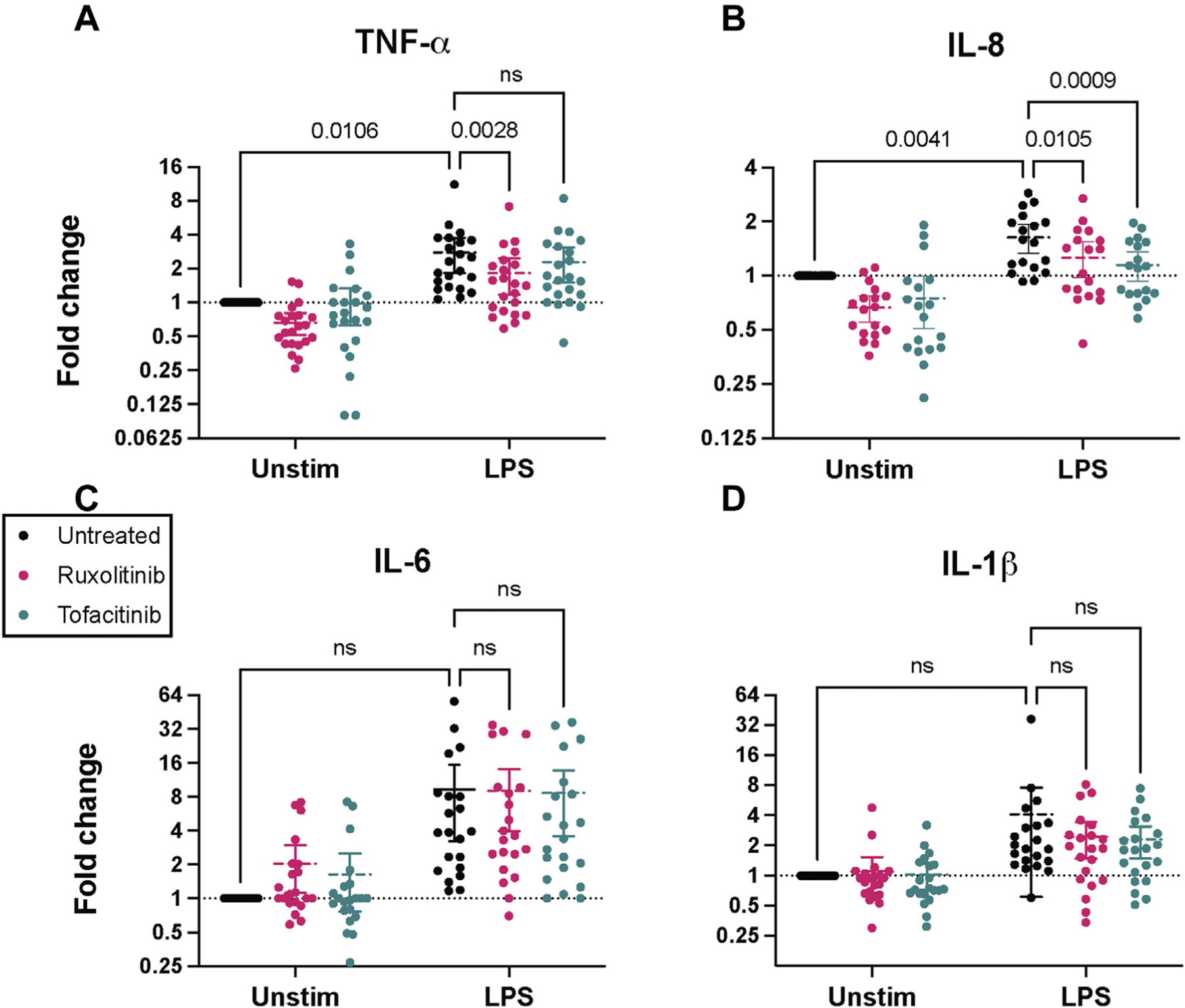
JAK inhibitors decrease inflammatory cytokine secretion from lamina propria CD11b^+^-enriched cell cultures. Lamina propria CD11b^+^-enriched cells were cultured in the presence of a JAK inhibitor, ruxolitinib or tofacitinib, and stimulated with LPS for 24 hours. Supernatant cytokine levels were measured via Luminex. Cytokine fold change for each condition was compared with unstimulated, untreated baseline average. Mean and 95% confidence intervals plus *P*-values from relevant 2-way ANOVA comparisons are shown (*P* ≤ .05 or ns = not significant). (A) TNF-*α* (n = 22), (B) CXCL8/IL-8 (n = 18), (C) IL-6 (n = 22), and (D) IL-1*β* (n = 18). ANOVA, analysis of variance.

Given that *JAK2* and the STAT3 pathway were significantly upregulated in anti-TNF-refractory ileal samples, we asked whether currently available JAK inhibitors could decrease inflammatory cytokine production in CD11b^+^ cells. We cultured CD11b^+^-enriched cells from patient biopsies and stimulated these cells with or without LPS, a potent bacterial activator of inflammation via TLR4.^38^ We co-cultured overnight with either tofacitinib, a UC-approved JAK1/JAK3 inhibitor, or ruxolitinib, a JAK1/JAK2 inhibitor. We then measured levels of 4 cytokines relevant to IBD pathogenesis^15^: TNF*α*, IL-1*β*, IL-6, and IL-8. Overall, LPS induced cytokine release across all samples, but samples treated with JAK inhibitors had decreased TNF*α* and IL-8 production (Figure 5A and B). This trend was more notable in ileal samples. IL-1*β* and IL-6 were not significantly affected by the JAK inhibitors (Figure 5C and D). We did not see any differences in the outcome whether cells came from inflamed or uninflamed samples. These findings provide evidence that JAK inhibitors can decrease TNF*α* and IL-8 production by phagocytes within the gut mucosa, at the direct site of inflammation in IBD.

### Differential Gene Expression in CD11b^+^ Cells Is Linked to eQTLs and Known IBD Risk Loci

Genomic sequencing data were available for 16 of the patients enrolled in our study (20 colon and 14 ileum samples) (Table 2). To further understand the regulation of gene expression in our RNA sequencing data, we used patient genomic data to identify expression quantitative trait loci (eQTLs), that is, genetic variants that affect gene expression. First, we selected a group of known eQTLs for the highest DE genes (log_2_ fold change ≥6) in our data sets. Using regression modeling, we found that one of these variants, rs846398 for *INHBA* (Inhibin beta A), demonstrated an eQTL effect in our colon samples (beta = −0.68, *P ≤* .05) (Table A7). This means that patients with this variant showed lower expression of *INHBA* in the colon. *INHBA* is a member of the TGF-*β* superfamily, and multiple studies have implicated *INHBA* expression as a prognostic marker for colorectal cancer.^39,40^

We then found 13 variants among 241 previously identified IBD risk loci^4^ that were classified as eQTLs for DE genes within our data set (Table A8). Using regression modeling, we found moderate evidence of an eQTL effect (*P ≤* .1) for 2 of these gene variants within our IBD samples: rs10797432 for *MMEL1* (membrane metalloendopeptidase-like 1) in the colon and ileum and rs3197999 for *MST1* (macrophage-stimulating 1) in the colon. These results were limited owing to small sample size, modest allele frequencies, or interactive effects of medication use and/or inflammatory state on gene expression. Nonetheless, our data suggest that these 2 established IBD risk variants may contribute to gene expression in CD11b^+^ cells based on location. In addition, we found that 26% (62 of 241) of IBD risk loci lie within 100 kilobases of a CD11b^+^ DE gene, 17% (40 of 241) within 50 kilobases, and 5% (11 of 241) within the DE gene itself (Tables A9–A11). Almost a third of these genes which are in close proximity to IBD risk loci are lncRNAs. This suggests that these IBD risk variants may be influencing expression related to tissue type or inflammatory state through pretranscriptional modifications, such as DNA-DNA interactions or alterations in chromatin state. Further study with a larger sample size would be necessary to determine their precise mechanism of influence.

## Discussion

Many new therapies are available to treat patients with IBD, but the general mucosal healing rates remain between 30% and 45%^41^ despite recent advances. If we hope to improve the proportion of patients that achieve mucosal healing and the best outcomes, we need to continue to find new targets for therapy and tailor these therapies appropriately. Most transcriptomic and functional studies have used either peripheral immune cell populations,^42,43^ which do not reflect intestinal inflammation, or whole tissue biopsies.^44–46^ Studies that have relied on tissue from surgical resections represent quite late-stage disease and may miss earlier targetable pathways.^13^ Interestingly, single-cell sequencing excludes specific cell types, such as neutrophils,^13^ which define active inflammation and endoscopic healing.^12^ Therefore, a strength of our study was enriching for relevant mucosal populations such as granulocytes (including neutrophils), macrophages, and dendritic cells. We took advantage of a powerful unbiased approach—RNA sequencing with bioinformatics—to look carefully at the transcriptomic profiles of these lamina propria phagocytes.

We found several surprises. The first is that the largest differences in CD11b^+^ cell gene expression were found between cells isolated from the colon vs the ileum. We confirmed with flow cytometry that there were no overt differences in the distribution of the CD11b^+^ cell types between locations. This may therefore speak to different functions of these cells in different environments. Indeed, CD11b^+^ cells derived from the colon had greater upregulation of immune pathways than those from the ileum. This observation may be due to the higher microbial burden in the colon compared with the ileum. In addition, many cancer genes and pathways were upregulated in the colon, suggesting that phagocytes may contribute to the microenvironment that promotes tumorigenesis in IBD.^26,38^ In contrast, metabolic pathways were upregulated in CD11b^+^ cells of the ileum, also likely in response to the ileal environment where nutrient absorption mainly takes place. Many of the altered metabolic pathways included known microbiota-produced metabolites, such as serotonin^20^ and bile acids,^21^ and could stem from IBD-linked dysbiosis. Co-analyzing microbiome and metabolomic data with gene expression data would enhance our understanding of their interplay within IBD.

Inflammatory cytokines and receptors, as well as known markers of inflammation, were consistently upregulated in inflamed samples. However, we found fewer differences between UC vs CD samples. These data suggest that lamina propria phagocytes are not the main contributor to the clinical phenotypic differences seen between these diseases. On the other hand, it does fit the observation that most therapies work for both UC and CD.

Regarding therapy, up to 40% of patients with IBD do not respond or lose response to anti-TNF biologics over time.^47^ Although characterization of primary nonresponse to anti-TNF has been studied,^31^ less is known about patients who have lost response. Ideally, gene expression could guide rational selection of medical therapy. Our study points to a novel approach by using lamina propria phagocytes as read-outs of mucosal immune responses. We identified genes and pathways that are activated in anti-TNF refractory patients and are targetable, such as *NTS*, *OSM*, *IL6*, *IL8*, inflammasome, toll-like receptor signaling, JAK-STAT, and TREM1 signaling. Inhibiting these genes or pathways upregulated in anti-TNF refractory patients could therefore be a possible standalone therapy or adjunct therapy to anti-TNFs. For example, TREM1 inhibitors have shown great promise in colitis mouse models^35^ and clinical trials for septic shock,^48^ but they have not yet been tested in patients with IBD. Therapies targeting OSM and OSMR are still in the conception phase.^49^ JAK inhibitors have already been approved for IBD,^50^ and others are in the pipeline.^24,51^ Our study provides functional data that JAK inhibition with tofacitinib (JAK1/JAK3) or ruxolitinib (JAK1/JAK2) inhibits LPS-induced cytokine production even in TNF-refractory samples. Recent clinical trials of ruxolitinib have been successful in treating inflammatory and autoimmune diseases such as rheumatoid arthritis and psoriasis.^52^ Pan-JAK inhibitors are also being developed with gut selectivity to reduce systemic toxicity.^53^ These data inform the response of patients to JAK inhibitors, including those refractory to other treatments.

In conclusion, our study on lamina propria–derived CD11b^+^ phagocytes identifies targetable genes/pathways and highlights the regional specialization of these cells. In addition, we provide evidence that JAK inhibitors alter cytokine production in gut-derived CD11b^+^ cells and evidence of eQTLs in CD11b^+^ cells. Our data suggest that highly DE genes such as *NTS* and *OSM*, as well as pathways such as JAK-STAT and TREM1 signaling, may be used to build precision medicine approaches in IBD. Future studies should further investigate these genes and pathways, as well as the many others in our data sets, to determine their clinical potentials.

## Methods

### Subjects, Sample Acquisition, and Ethics Statements

The use of human samples for this study was approved by the University of Miami Institutional Review Board (IRB ID: 20081100). Endoscopic biopsy samples were obtained from patients diagnosed with CD or UC who provided informed consent for the IBD Clinical Phenotype Database and Specimen Collection. Each sample contained 4–6 adjacent mucosal biopsies of approximately 3 mm diameter. One hundred eleven samples were obtained from the terminal ileum, ascending colon, or sigmoid colon from a total of 54 patient subjects. Our sample database was annotated with respect to inflammation based on endoscopy and histology. Samples that were endoscopically uninflamed but histologically inflamed were categorized as inflamed. Deidentified demographic and clinical data for study subjects were also provided for analysis (Table 1). All authors had access to the study data and reviewed and approved the final manuscript before submission.

### CD11b^+^ Cell Isolation From Mucosal Biopsies

On collection, mucosal biopsies were placed in Hypo-Thermosol solution (MilliporeSigma, St. Louis, MO) at 4 °C. Intestinal epithelial cells were then depleted by incubation in Dulbecco’s Modified Eagle Medium with 10 mM dithiothreitol, 0.5 mM Ethylenediamine tetraacetic acid, and 10,000 units/mL penicillin/streptomycin for 20 minutes. Lamina propria cells were dissociated by digesting tissue in DMEM with 250 *μ*g/mL Liberase (MilliporeSigma) and 10 *μ*g/mL DNase I (Lucigen Corporation, Middleton, WI) at 37 °C for 20 minutes. Digested tissue was further mechanically dissociated by pipetting to obtain a single-cell suspension and filtered using a 70-*μ*m strainer. Cell suspensions were labeled with 20 *μ*L of CD11b MicroBeads per 10^7^ cells (Miltenyi Biotec, Auburn, CA) for positive selection via Magnetic-Activated Cell separation (MACs) on LS columns (Miltenyi Biotec). After sorting, cells were counted via a hemocytometer and added to RPMI media for culturing, FACS buffer for flow cytometry analysis, or RLT buffer (Qiagen, Germantown, MD) with 10% *β*-mercaptoethanol for RNA isolation.

### Luminex Cytokine Assay

CD11b^+^-enriched cells (10,000–20,000 cells/well) from 27 samples were cultured in RPMI-1640 media supplemented with 10% fetal bovine serum, 10,000 units/mL of pen/strep, 1% GlutaMAX (Thermo Fisher Scientific, Waltham, MA), and 5 ng/mL GM-CSF. Six different experimental conditions were tested per sample: (1) unstimulated cells; (2) stimulated with 10 *μ*g/mL LPS; (3) 1 *μ*M^11,54^ ruxolitinib (InvivoGen, San Diego, CA); (4) 1 *μ*M tofacitinib (Invivogen); (5) 10 *μ*g/mL LPS 1 mM ruxolitinib; and (6) 10 *μ*g/mL LPS + 1 *μ*M tofacitinib. After overnight incubation, supernatants were collected for cytokine quantification by Luminex using a Human High Sensitivity Cytokine customized premixed magnetic bead panel (R&D Systems, Minneapolis, MN) for the following: IL-1 *β*/IL-1F2, IL-6, IL-8/CXCL8, and TNF. The median fluorescence intensity data were analyzed using MILLIPLEX Analyst Software V.3.5 (MilliporeSigma), and concentrations were calculated in pg/mL in relation to an R&D standard.

### Flow Cytometry and CD11b^+^ Cell Phenotyping

Percent cell yield and phenotyping of the CD11b^+^-enriched cells were determined from 10 inflamed and 20 uninflamed samples by flow cytometry with a panel of 11 antibodies (Table A1, Figure A1, and Figure A2). Flow cytometry was performed on the BD FACSAria IIu (BD Bioscience, San Jose, CA) at the University of Miami Flow Cytometry Shared Resource and analyzed using FCS Express 7 (De Novo Software, Los Angeles, CA).

### RNA Extraction and Next-Generation Sequencing

RNA was isolated from 60 CD11b^+^ cell–enriched samples with the RNeasy Mini Kit (QIAGEN, Germantown, MD) and sequenced at the University of Miami Onco-Genomics Shared Resource. Libraries were prepared using the KAPA RNA HyperPrep Kit with RiboErase (HMR; Roche Life Science, Wilmington, MA) and sequenced on a 200-bp paired-end run with the Illumina NovaSeq 6000 S1 (100 cycles; 1.6 billion flow cells). Sixty barcoded libraries were sequenced across 2 lanes.

### Quantitative Polymerase Chain Reaction

Hundred nanogram of RNA from a selection of anti-TNF–treated ileum samples (n = 7) was retro-transcribed using the PrimeScript RT reagent kit (Takara Bio USA). The resulting cDNA was amplified on a LightCycler 480 II instrument (Roche Life Science) with selected primers (Table A2) using the SYBR Premix Ex Taq (Takara Bio USA). Relative messenger RNA expression was calculated by means of the change in the cycle threshold (ΔΔCt) and normalized to the geometric mean of the housekeeping genes *ACTB* and *GUSB*.

### Genomic Sequencing

Sixteen patients within the study had previously provided samples for whole-genome sequencing (WGS) or Global Screening Array (GSA). All DNA samples were processed and sequenced or genotyped at the Broad Institute of Harvard and MIT using standard protocols.

### Statistical Methods and Bioinformatics

Statistical analyses were performed using the R software (https://www.r-project.org/) and Prism 8.0 (GraphPad Software, San Diego, CA). For comparing cell counts and percentages between uninflamed and inflamed samples, an unpaired t-test was used for normally distributed data and the Mann-Whitney U test for non-normally distributed data. For Luminex cytokine release assays, median fluorescence intensity for each condition was compared with condition 1 (unstimulated and untreated cells) to obtain a fold change value. Results were analyzed via 2-way analysis of variance, with *P ≤* .05 considered statistically significant.

For RNA sequencing, 4.18 billion paired-end reads (2.09 billion clusters) were generated, and 472 million paired-end reads (236 million clusters) were unindexed. A total of 58 samples passed quality check with >78% uniquely aligned reads, which were mapped to the human genome (GRCh38) using the STAR (Ver. 2.5.0) aligner.^55^ Raw counts were generated based on Ensembl genes (GENCODE, version 26) with featureCounts (version 1.5.0)^56^. The RNA sequencing data were deposited in NCBI’s Gene Expression Omnibus^57^ and will be accessible to the public through GEO Series accession number GSE183620 (https://www.ncbi.nlm.nih.gov/geo/query/acc.cgi?acc=GSE183620) on September 02, 2024. DESeq2 (version 1.28.1)^58^ was used for differential expression analysis, adjusting for intestinal epithelial cell enrichment scores as a covariate variable to ensure significant differences in gene expression levels were not due to differing amounts of epithelial cells in each sample. Epithelial enrichment scores were estimated using the xCell software.^59^ Samples were compared based on location, inflammation status, IBD type, and/or treatment. Genes with a false discovery rate (adjusted *P*-value) ≤ 0.05 were considered to be statistically significant. Pathway analyses were performed using Metascape (http://metascape.org)^60^ and IPA (QIAGEN, https://www.qiagenbioinformatics.com/products/ingenuitypathway-analysis).^61^ Effect of confounding variables was assessed via the Wilcoxon test and Fisher’s exact test for between-group comparisons of categorical and continuous variables, respectively.

For WGS data, sequence variants were jointly called across **~**1090 samples via the Genome Analysis Toolkit Best Practices for germline variants.^62^ Genomic variants from GSA were jointly called with a larger set of **~**900 samples and imputation performed using TOPMED.^63^ Quality control was implemented to ensure no batch effects between WGS and GSA samples.

For eQTL analysis, the Genotype-Tissue Expression Project (GTEx) was used to identify eQTLs in whole blood, sigmoid colon, transverse colon, or small intestine for DE genes in our RNA sequencing data. A linear regression model of the GTEx eQTL minor allele count (0, 1, or 2) against TPM (transcripts per kilobase per million mapped reads) was performed for high DE genes (log2 fold change ≥6). Inflammatory status, medication use, and ethnicity were included as covariates in the model. For this analysis, only eQTLs with Genome Aggregation Database (gnomAD) European and Hispanic population frequencies ≥0.30 were included to maintain ample power.^64^ GTEx eQTLs were then further selected among the top associated variants within 241 known IBD risk loci.^4^ eQTL analysis was performed again as described on DE genes with at least 0.01 TPM in at least 90% of the samples. For both regression models, an eQTL effect was reported for variants with a *P*-value ≤.1. Finally, known IBD risk variants were mapped to directly within DE genes (log2 fold change ≥1.5) as well as within 50 or 100 kilobases upstream or downstream of these genes.

## Supplementary Material

Figure Legends and Tables A1–A3

Tables A4–A11

Figure A6

Figure A5

Figure A4

Figure A3

Figure A2

Figure A1

## Figures and Tables

**Table 1.** Demographics and Baseline Characteristics

Total participants enrolled	N = 54
Sex	
Male	27 (50%)
Female	27 (50%)
Ethnicity	
Hispanic	24 (44%)
Non-Hispanic	30 (56%)
Race	
White	50 (93%)
African American	1 (2%)
Asian	1 (2%)
Other	2 (4%)
Smoking status at diagnosis	
Nonsmokers	41 (76%)
Ex-smokers	8 (15%)
Smokers	5 (9%)
Age at diagnosis (median)	23 y
Age at enrollment (median)	37 y
Current BMI (median)	25.5
Medications	
Currently no medication	9 (16%)
Current use of aminosalicylates	16 (30%)
Past use of aminosalicylates	38 (70%)
Current use of steroids	8 (15%)
Past use of steroids	38 (70%)
Current use of immunomodulators	6 (11%)
Past use of immunomodulators	28 (52%)
Current use of biologics	32 (59%)
Anti-TNF agents	20 (37%)
Anti-integrin	8 (15%)
Anti-IL-12/23	4 (7%)
Past use of biologics	22 (41%)
1 anti-TNF agent	13 (24%)
2 anti-TNF agents	6 (11%)
3 or more anti-TNF agents	2 (4%)
Other	5 (9%)
UC	23 (43%)
Location of UC	
Pancolitis	11 (20%)
Left sided	12 (22%)
History of gastrointestinal surgeries	0 (0%)
CD	31 (57%)
Location of CD	
Ileum only	11 (20%)
Ileum and colon	15 (28%)
Colon only	5 (9%)
History of gastrointestinal surgeries	12 (22%)

BMI, body mass index.

**Table 2.** Sample Information

	RNA sequencing	Flow cytometry	Luminex	qPCR	eQTL analysis
Total biopsies	58	30	27	7	34
Disease					
UC	37 (64%)	15 (50%)	11 (41%)	5 (71%)	28 (82%)
CD	21 (36%)	15 (50%)	16 (59%)	2 (29%)	6 (18%)
Tissue location					
Ileum	26 (45%)	12 (40%)	14 (52%)	7 (100%)	14 (41%)
Colon	32 (55%)	18 (60%)	13 (48%)	0 (0%)	20 (59%)
Inflammation (by histology)					
Inflamed	20 (34%)	10 (33%)	15 (56%)	3 (43%)	13 (38%)
Uninflamed	38 (66%)	20 (67%)	12 (44%)	4 (57%)	21 (62%)
Current medications					
Biologics	31 (53%)	22 (73%)	12 (44%)	7 (100%)	15 (44%)
Anti-TNF	15 (26%)	17 (57%)	8 (30%)	7 (100%)	11 (32%)
Anti-integrin	12 (21%)	5 (17%)	3 (11%)	-	3 (9%)
Anti-IL12/IL23	4 (7%)	-	1 (4%)	-	1 (3%)
Non-biologics	34 (58%)	15 (50%)	13 (48%)	-	21 (6%)
Steroids	11 (19%)	-	5 (19%)	-	4 (12%)
Mesalamines	20 (34%)	12 (40%)	7 (26%)	-	17 (50%)
Thiopurines	5 (9%)	3 (10%)	4 (15%)	-	2 (6%)

## Abbreviations used in this paper:

CD: Crohn’s disease
DE: differentially expressed
eQTLs: expression quantitative trait loci
GSA: Global Screening Array
GTEx: Genotype-Tissue Expression Project
IBD: inflammatory bowel disease
IL: interleukin
IPA: Ingenuity Pathway Analysis
lncRNA: long noncoding RNA
JAK: janus kinase
JAK-STAT: janus kinase-signal transducer and activator of transcription
LPS: lipopolysaccharide
TNF: tumor necrosis factor
UC: ulcerative colitis
WGS: whole-genome sequencing
