# Supplementary material for: Lamina Propria Phagocyte Profiling Reveals Targetable Signaling Pathways in Refractory Inflammatory Bowel Disease
Source: Gastro Hep Adv. Author manuscript; Available in PMC 2022 Sep 2. (PMC9438737; doi:10.1016/j.gastha.2022.01.005)
Supplement: Figure Legends and Tables A1–A3 [file NIHMS1827843-supplement-Figure_Legends_and_Tables_A1_A3.docx]

**Supplementary figure legends:**

**Supplementary Figure S1. Flow cytometry gating scheme.** Live, single cells were gated in P1-P3 before being divided into various cell populations. A representative unenriched mucosal biopsy sample is shown so that certain low abundance populations (e.g. Epcam/CD326^+^ intestinal epithelial cells) can be appreciated. Related to Figure 1B and 1C.

**Supplementary Figure S2.** Flow cytometry fluorescence-minus-one for each marker performed on unenriched samples. Related to Figure 1B and 1C.

**Supplementary Figure S3.** Average number of cells per sample and average percentage of each cell type per sample based on flow cytometry for uninflamed and inflamed samples. Related to Figure 1A and 1C.

**Supplementary Figure S4.** Expression of select DE genes in anti-TNF-treated ileum samples, inflamed vs uninflamed, were validated by qPCR (n = 7). All eight genes tested followed the same expression trend as RNA sequencing. Related to Figure 3G.

**Supplementary Figure S5.** TRRUST upstream regulators based on DE genes (adjusted *P*-value ≤ 0.05) from anti-TNF-treated inflamed ileum (vs anti-TNF-treated uninflamed ileum). Related to Figure 3G.

**Supplementary Figure S6.** Ingenuity Pathway Analysis (IPA) network visualization of the STAT3 pathway used to create Figure 4B.

**Supplementary tables:**

**Supplementary Table S1. Flow cytometry panel for CD11b^+^ cell phenotyping**

| **Marker** | **Color/Format** | **Host/Target** | **Isotype** | **Clone** | **Company** | **Catalog** |
| --- | --- | --- | --- | --- | --- | --- |
| CD45 | APC-Cy7 | Mouse anti-Human | IgG1 | 2D1 | BioLegend [877-246-5343] | 368516 |
| CD11b | BB515 | Mouse anti-Human | IgG1 | ICRF44 (also known as 44) | BD Biosciences [877.232.8995] | 564518 |
| CD66 | APC | Mouse anti-Human | IgG2a | CD66a-B1.1 | Thermo Fisher Scientific [1 800 955 6288] | 17-0668-42 |
| CD56 | PE-Cy5 | Mouse anti-Human | IgG1 | B159 | BD Biosciences [877.232.8995] | 561904 |
| CD14 | Brilliant Violet 650 | Mouse anti-Human | IgG2a | M5E2 | BioLegend [877-246-5343] | 301836 |
| CD1c | Brilliant Violet 421 | Mouse anti-Human | IgG1 | L161 | BioLegend [877-246-5343] | 331526 |
| CD3 | BUV496 | Mouse anti-Human | IgG1 | UCHT1 | BD Biosciences [877.232.8995] | 564810 |
| CD19 | PE-eFluor 610 | Mouse anti-Human | IgG1 | HIB19 | eBioscience (Thermo Fisher Scientific)  [1 800 955 6288] | 61-0199-42 |
| CD326 | Brilliant Violet 605 | Mouse anti-Human | IgG2b | 9C4 | BioLegend [877-246-5343] | 324224 |
| S100A4 | PE | Mouse anti-Human | IgG1 | NJ-4F3-D1 | BioLegend [877-246-5343] | 370004 |
| CD31 | PE-Cy7 | Mouse anti-Human | IgG1 | WM59 | BioLegend [877-246-5343] | 303118 |

**Supplementary** **Table S2. Quantitative Polymerase Chain Reaction (qPCR) primers**

| **Gene** | **Sense** | **Primer** | **GenBank Accession** | **PMID** |
| --- | --- | --- | --- | --- |
| CXCL5 | F | AGCTGCGTTGCGTTTGTTTAC | NM_002994 |  |
| CXCL5 | R | TGGCGAACACTTGCAGATTAC | NM_002994 |  |
| CCL25 | F | GGCCCTCATGCTGTAAAGAAG | NM_005624 |  |
| CCL25 | R | TGCTGATGGGATTGCTAAACTT | NM_005624 |  |
| IL1B | F | TACCTGTCCTGCGTGTTGAA |  | 29197519 |
| IL1B | R | TCTTTGGGTAATTTTTGGGATCT |  | 29197519 |
| S100A9 | F | GGAATTCAAAGAGCTGGTGC |  | 22304731 |
| S100A9 | R | TCAGCATGATGAACTCCTCG |  | 22304731 |
| OSM | F | CACAGACTGGCCGACTTAGAG | NM_020530 |  |
| OSM | R | AGTCCTCGATGTTCAGCCCA | NM_020530 |  |
| NTS | F | TGCTTTAGATGGCTTTAGCTTGG | NM_006183 |  |
| NTS | R | TTCCTGGATTAACTCCCAGTGT | NM_006183 |  |

**Supplementary Table S3. Effect of potential confounder variables in differential gene expression analysis.** Age, gender, race, and disease years are not associated with tissue location, either colon or ileum (*P* ≤ 0.05). Therefore, these variables are not confounders in our differential expression analysis that tests association between tissue location and gene expression.

|  | **P-value** |
| --- | --- |
| **Tissue location vs Gender** | 0.3028205 |
| **Tissue location vs Race** | 1 |
| **Tissue location vs Age** | 0.9784796 |
| **Tissue location vs Disease years** | 0.9870884 |
