# Supplementary figures and images for: Lamina Propria Phagocyte Profiling Reveals Targetable Signaling Pathways in Refractory Inflammatory Bowel Disease
Source: Gastro Hep Adv. Author manuscript; Available in PMC 2022 Sep 2. (PMC9438737; doi:10.1016/j.gastha.2022.01.005)

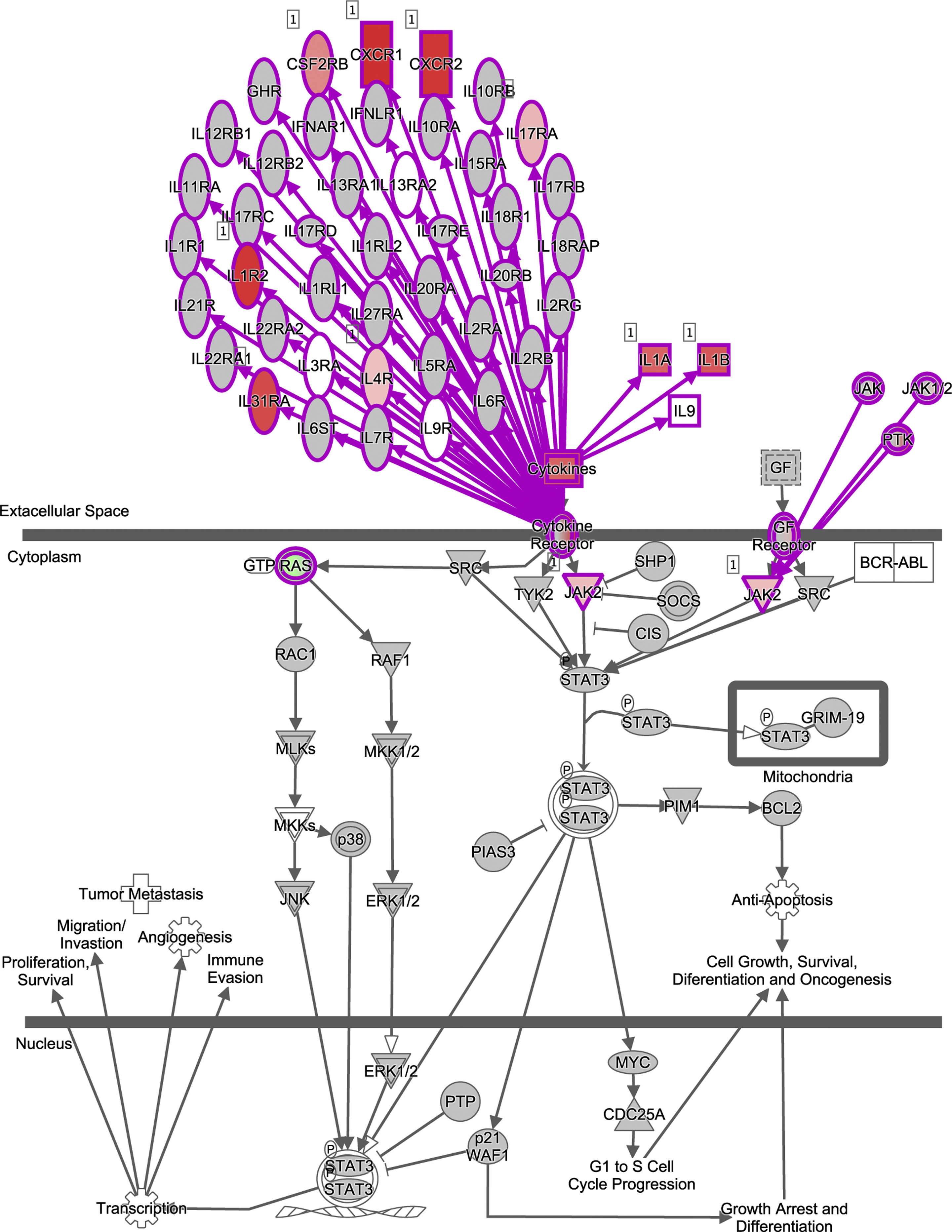

Supplement: Figure A6 [file NIHMS1827843-supplement-Figure_A6.jpg]

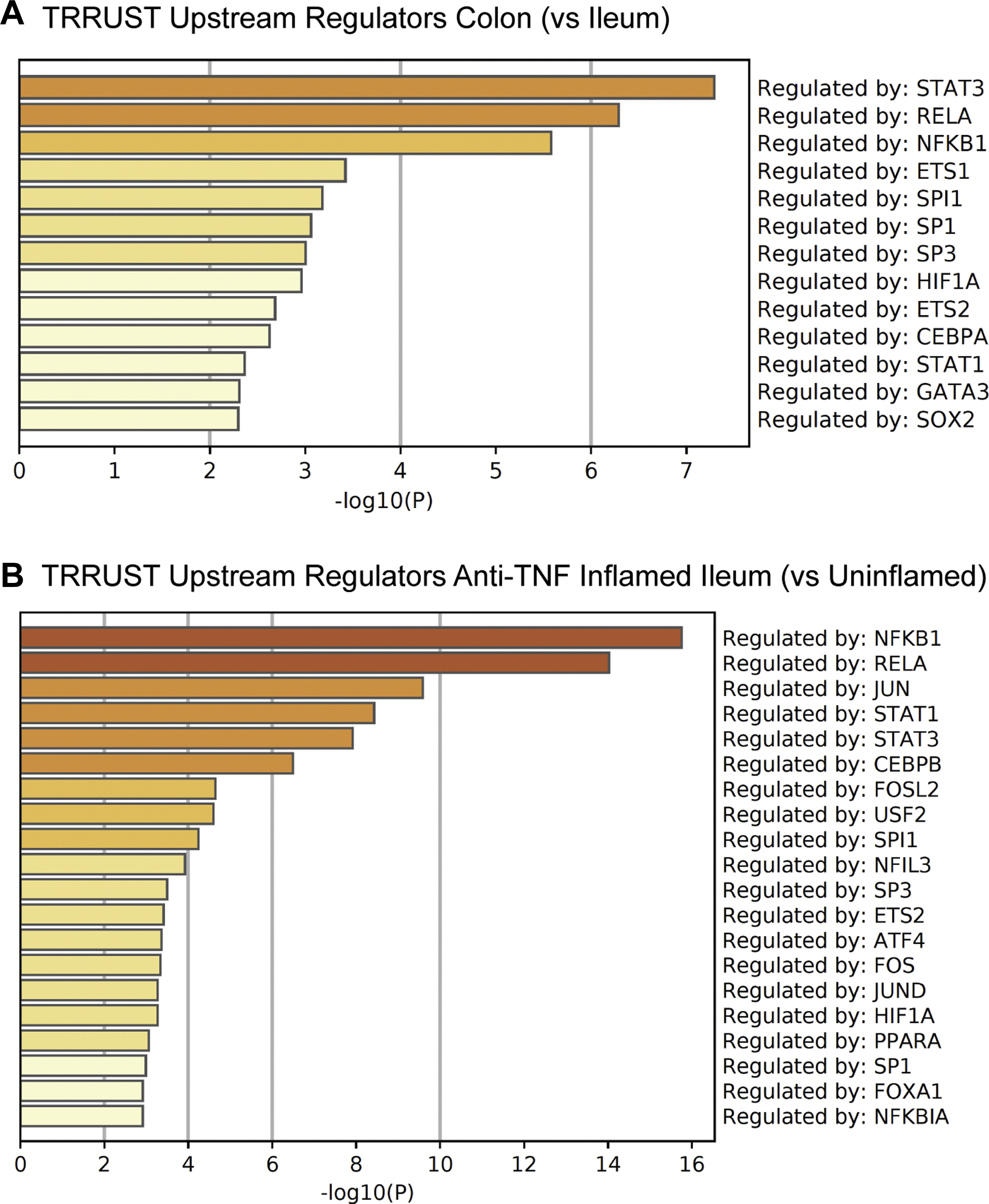

Supplement: Figure A5 [file NIHMS1827843-supplement-Figure_A5.jpg]

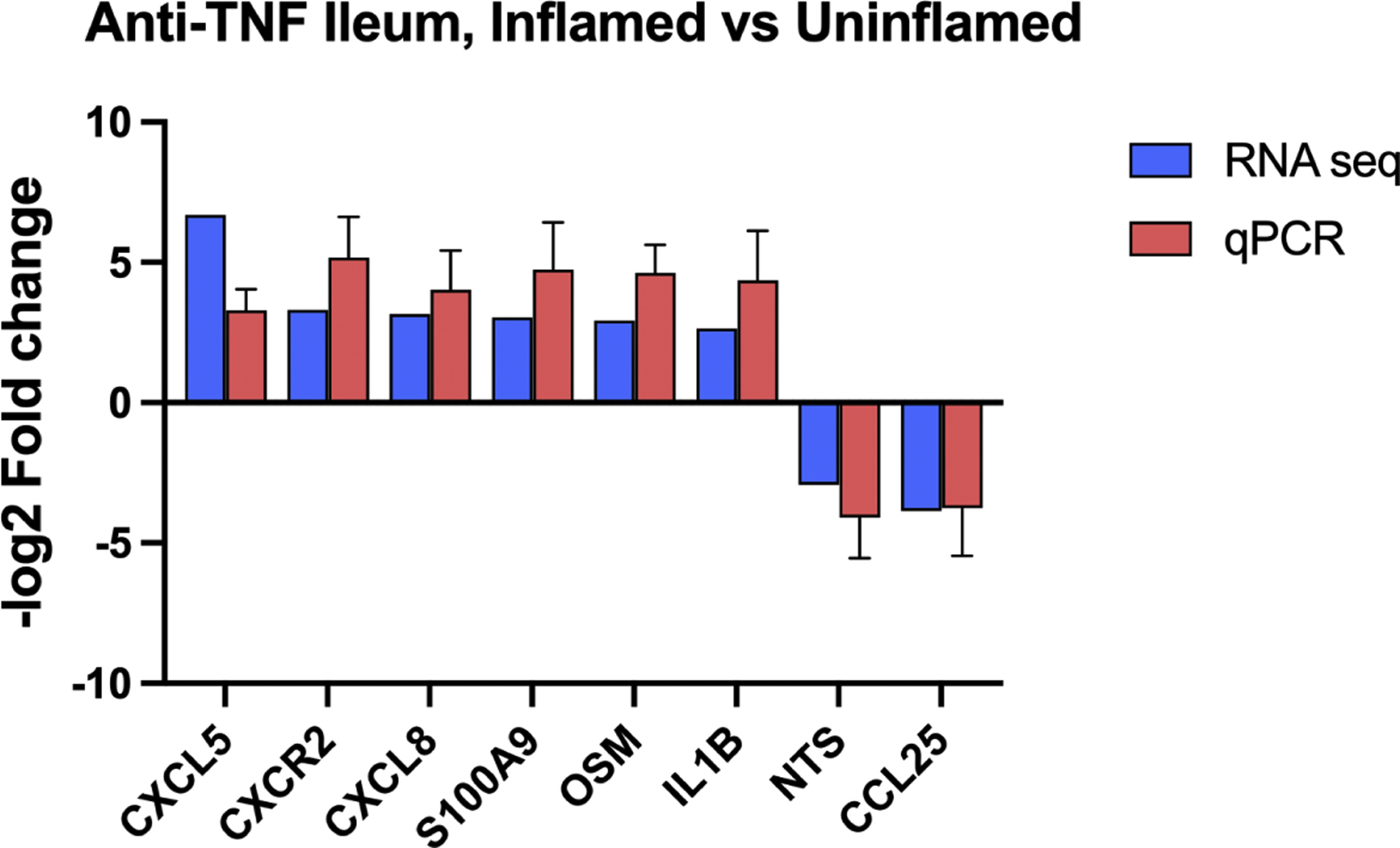

Supplement: Figure A4 [file NIHMS1827843-supplement-Figure_A4.jpg]

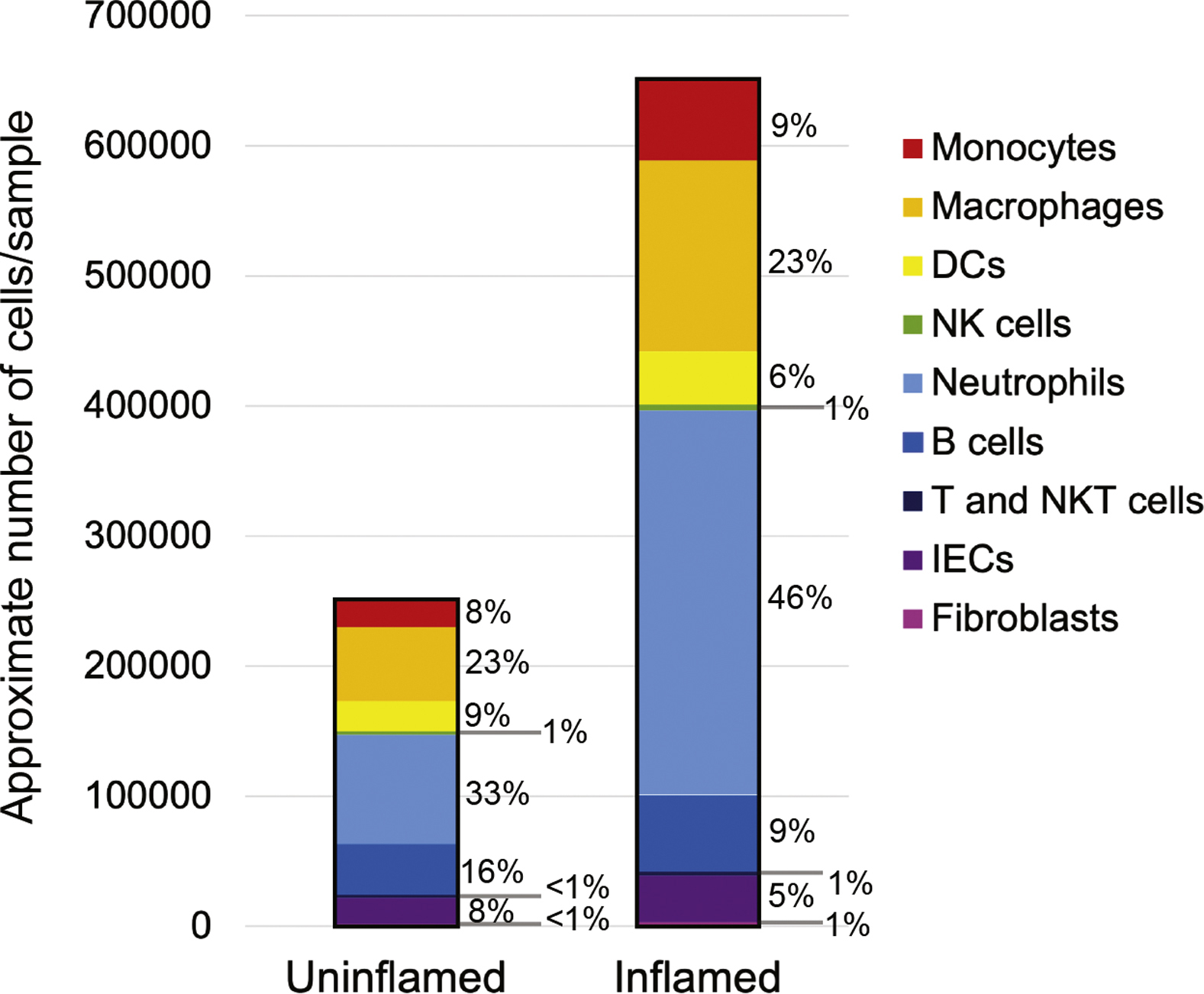

Supplement: Figure A3 [file NIHMS1827843-supplement-Figure_A3.jpg]

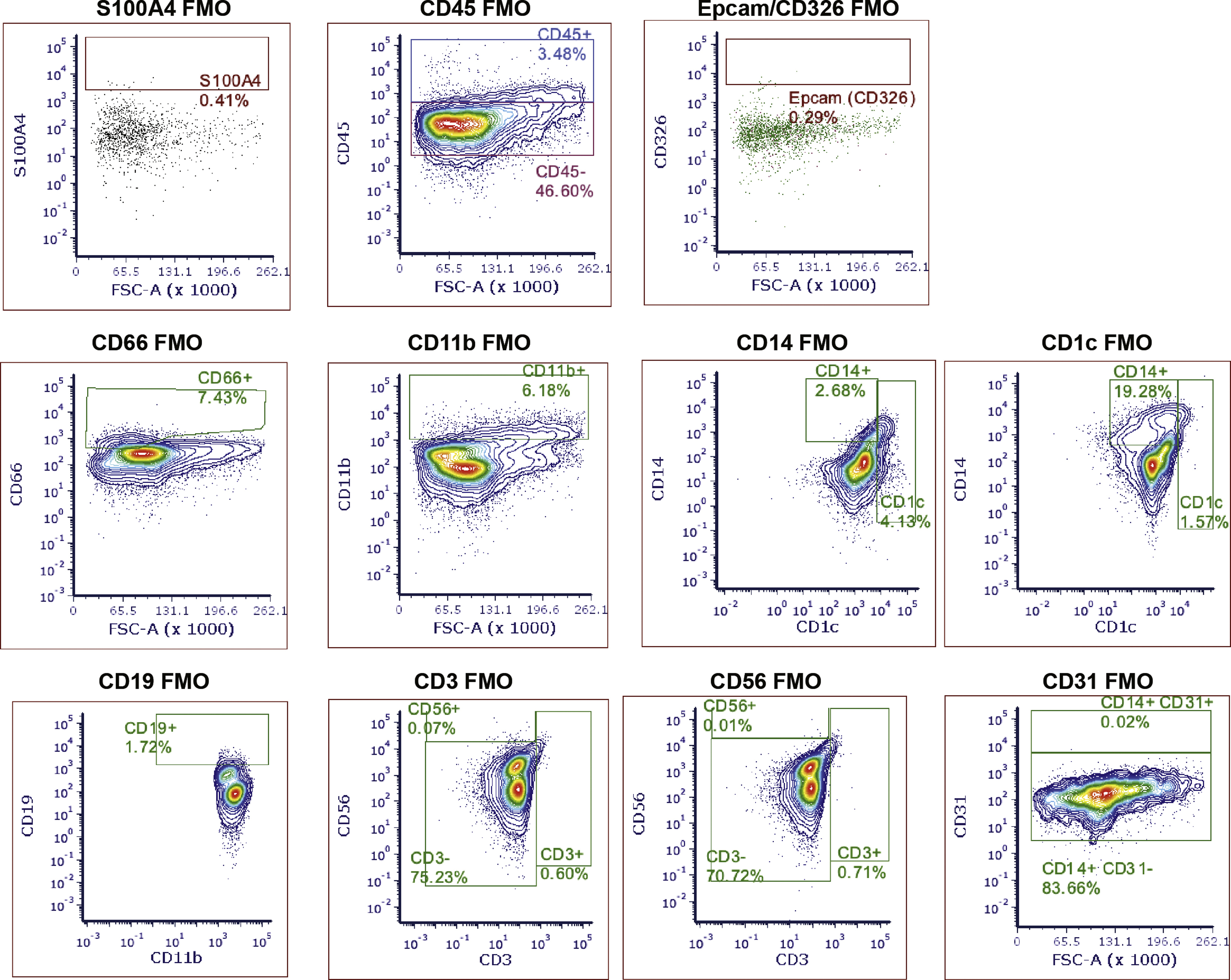

Supplement: Figure A2 [file NIHMS1827843-supplement-Figure_A2.jpg]

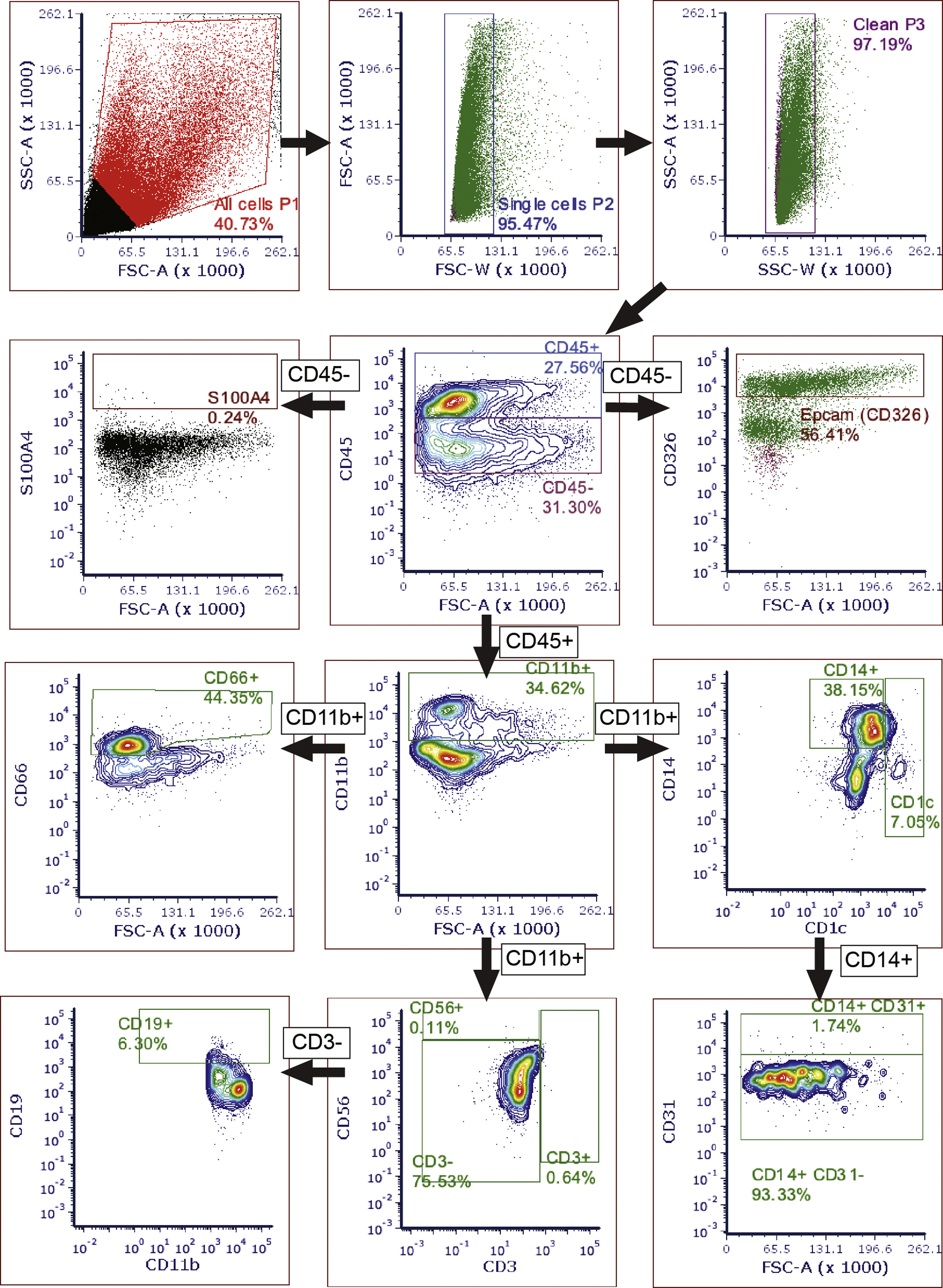

Supplement: Figure A1 [file NIHMS1827843-supplement-Figure_A1.jpg]
